# Rutamarin: Efficient Liquid–Liquid Chromatographic Isolation from *Ruta graveolens* L. and Evaluation of Its In Vitro and In Silico MAO-B Inhibitory Activity

**DOI:** 10.3390/molecules25112678

**Published:** 2020-06-09

**Authors:** Ewelina Kozioł, Simon Vlad Luca, Hale Gamze Ağalar, Begüm Nurpelin Sağlık, Fatih Demirci, Laurence Marcourt, Jean-Luc Wolfender, Krzysztof Jóźwiak, Krystyna Skalicka-Woźniak

**Affiliations:** 1 Independent Laboratory of Natural Products Chemistry, Department of Pharmacognosy, Medical University of Lublin, 20-093 Lublin, Poland; ewelinakoziol@umlub.pl; 2 Department of Pharmacognosy, Grigore T. Popa University of Medicine and Pharmacy Iasi, 700115 Iasi, Romania; simon-vlad.v.luca@d.umfiasi.ro; 3 Biothermodynamics, TUM School of Life and Food Sciences Weihenstephan, Technical University of Munich, 85354 Freising, Germany; 4 Department of Pharmacognosy, Faculty of Pharmacy, Anadolu University, 26470 Eskisehir, Turkey; ecz.halegamze@gmail.com (H.G.A.); bnsaglik@anadolu.edu.tr (B.N.S.); demircif@gmail.com (F.D.); 5 Faculty of Pharmacy, Eastern Mediterranean University, Famagusta 99628, Cyprus; 6 Institute of Pharmaceutical Sciences of Western Switzerland, IPSWS, University of Geneva, CMU, 1211 Geneva 4, Switzerland; laurence.marcourt@unige.ch (L.M.); Jean-Luc.Wolfender@unige.ch (J.-L.W.); 7 Department of Biopharmacy, Medical University of Lublin, 20-093 Lublin, Poland; krzysztof.jozwiak@umlub.pl

**Keywords:** monoamineoxidase, coumarins, Parkinson’s disease, Alzheimer’s disease, liquid–liquid chromatography, countercurrent chromatography

## Abstract

Naturally occurring coumarins are a group of compounds with many documented central nervous system (CNS) activities. However, dihydrofuranocoumarins have been infrequently investigated for their bioactivities at CNS level. Within the frame of this study, an efficient liquid–liquid chromatography method was developed to rapidly isolate rutamarin from *Ruta graveolens* L. (Rutaceae) dichloromethane extract (DCM). The crude DCM (9.78 mg/mL) and rutamarin (6.17 µM) were found to be effective inhibitors of human monoamine oxidase B (*h*MAO-B) with inhibition percentages of 89.98% and 95.26%, respectively. The inhibitory activity against human monoamine oxidase A (*h*MAO-A) for the DCM extract was almost the same (88.22%). However, for rutamarin, it significantly dropped to 25.15%. To examine the molecular interaction of rutamarin with *h*MAO- B, an *in silico* evaluation was implemented. A docking study was performed for the two enantiomers (*R*)-rutamarin and (*S*)-rutamarin. The (*S*)-rutamarin was found to bind stronger to the *h*MAO-B binging cavity.

## 1. Introduction

Neurodegenerative diseases are a burning issue of our times. Between 2000 and 2017, the number of deaths from Alzheimer’s disease (AD) in the United States increased by 145%. Among people aged 70, 61% of those with AD are expected to die before age 80, compared with 30% of people without AD [1]. On the other side, the World Health Organization (WHO) reports that 10–14 out of 100,000 people develop Parkinson’s disease (PD) every year. The mortality of PD is still elevated, despite the therapy with L-dopa and other novel treatment strategies, including invasive treatments. Thus, it is important to investigate different drug targets and drug candidates with potential use in the treatment of neurodegenerative diseases.

Monoamine oxidases (MAOs) are a known drug target for different central nervous system (CNS) disorders. MAOs are mammalian flavoenzymes bound to the outer mitochondrial membrane and occur in two isoforms A and B (MAO-A and MAO-B) responsible for catecholamines and serotonin catabolism. Both isoforms are involved in the etiology of various neurological disorders, like depression, AD and PD. Norepinephrine, epinephrine and dopamine are preferred substrates for MAO-A; moreover, this isoform also catalyzes the degradation of serotonin [2,3]. Therefore, MAO-A inhibitors, like hydrazine derivatives or moclobemide, are used in the pharmacotherapy of depression. On the other hand, higher levels of MAO-A and MAO-B are correlated with an increased level of neurotoxic metabolites as well as with neuronal loss and generation of plaque-associated glia [3]. Both forms of the enzyme take part in producing 3,4-dihydroxyphenylglycolaldehyde and 3,4-dihydroxyphenylacetaldehyde, metabolites known as inducers of neuronal apoptosis [4]. Additionally, hydrogen peroxide (H_2_O_2_), a side product of MAO catalysis, is relevant in the generation of toxic reactive oxygen species (ROS) in mitochondria; MAO-B inhibitors, like selegiline(*L*-deprenyl) or rasagiline, are used in combination with L-dopa to prevent its catabolism and increase the central dopamine levels [2,5]. Both MAO-A and MAO-B inhibitors reduce the oxidative stress produced by these enzymes and exert general neuroprotective effects [2].

In recent years, interest in coumarin-based compounds as potential molecular entities has increased. Taking into account the multidirectional action of coumarins on the CNS, both naturally occurring coumarins and their synthetic derivatives are promising candidates for future drugs. Their activity against both MAO isoforms has recently emerged a particular interest [6,7,8]. Knowing that commonly occurring coumarins, like bergapten, psoralen, xanthotoxin, praeruptorin A, auraptene [9,10] and rare derivatives, like lacinartin or monankarins A-F [11,12], were identified as MAO inhibitors, the activity of rutamarin, a linear dihydrofuranocoumarin, against *h*MAO-A and *h*MAO-B was evaluated in the current study. To date, data about its activity towards CNS are limited.

*Ruta graveolens* L. (common rue, Rutaceae) is a very well-known source of rutamarin and other specialized metabolites with interesting properties. For instance, the essential oil (prevalent in ketones, mainly 2-nonanone and 2-undecanone) is used as an environmentally friendly food preservative. Furthermore, incorporation of *R. graveolens* L. essential oil into edible chitosan coating increased the stability of Guava fruits for at least 12 days at room temperature [13,14,15]. Aside from essential oils and coumarins, *R. graveolens* L. also contains furoquinoline alkaloids and acridone derivatives, like arborine, graveoline, 1,4-dihydroxy-2,3-dimethoxy-*N*-methylacridone, 1-hydroxy-3-methoxy-*N*-methylacridone and *N*-methyl-4-methoxy-2-quinolone. These constituents were found to be able to inhibit the photosynthesis process, acting as plant growth inhibitors, which may be a valuable tool in the development of new classes of herbicides [16].

The aim of this study was to develop an efficient liquid–liquid chromatography method to isolate high-purity rutamarin from *Ruta graveolens* L. Rutamarin was further subjected to an in vitro study that that evaluated its *h*MAO-A and *h*MAO-B inhibitory activity and an in silico study that assessed the molecular interactions of rutamarin with *h*MAO-B.

## 2. Results and Discussion

### 2.1. Phytochemical Study: Liquid–Liquid Chromatography Isolation of Rutamarin

**Figure 1:**
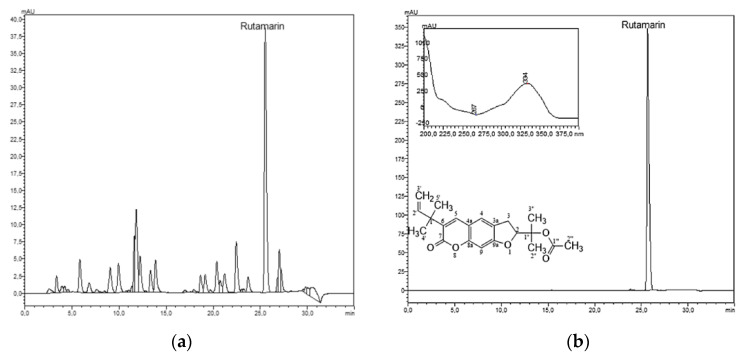
HPLC–DAD chromatograms of (**a**) crude dichloromethane extract of *Ruta graveolens* L. and (**b**) isolated rutamarin (λ=335 nm).

Rutamarin has been previously isolated from different parts (roots, leaves, aerial parts, fruits or stems) of *Ruta* sp., namely *R. angustifolia* [17], *R. chalapensis* [18] or *R. graveolens* [19]. Nevertheless, the purification of rutamarin has so far only been carried out by conventional chromatographic techniques (column chromatography or preparative HPLC) which are known to be time consuming, use large amounts of organic solvents and give low sample recoveries due to adsorption onto the solid support [20]. Therefore, in the current study, support-free liquid–liquid chromatography (LLC), commonly associated with countercurrent chromatography (CCC) or centrifugal partition chromatography (CPC), was evaluated as an alternative separation technique for the purification of rutamarin. As suggested by its name, both phases are liquid, one serving as the mobile phase and the other as the stationary phase, which is retained in specially designed columns with the help of a centrifugal field [21]. As there is no solid stationary phase, sample denaturation and adsorption-related processes are avoided, with maximum sample recovery. Short analysis times, high-loading capacities and reduced solvent consumptions make the technique economic and environmentally friendly [20]. The first step in any LLC separation is the selection of the biphasic solvent system; for this, the partition coefficient of the target component can be used as screening parameter. *P_rut_* was calculated by dividing the concentration of rutamarin in the stationary phase (upper phase) to that in the mobile phase (lower phase). Consequently, three different ratios of *n*-hexane, ethyl acetate, methanol and water (HEMWat) (5/2/5/2, 3/1/3/1 and 4/1/4/1, *v*/*v*/*v*/*v*) have been evaluated for their *P_rut_* values in regard to rutamarin (*P_rut_* = 1.39, 1.10 and 0.77, respectively). Taking into consideration that the partition coefficient of a target analyte should preferably be within the range 0.4–2.5 [21], it can be presumed that these three HEMWat mixtures can be used for the one-step purification of rutamarin, with more or less similar outcomes. Thus, parallel batch separations, each time with 100 mg of crude dichloromethane extract of *R. graveolens* L. (Figure 1), afforded 2.84 mg rutamarin (~99.0%) in 43 min after elution with HEMWat 5/2/5/2 (*v*/*v*/*v*/*v*), 2.39 mg rutamarin (~99.5%) in 24 min after elution with HEMWat 3/1/3/1 (*v*/*v*/*v*/*v*) and 2.13 mg rutamarin (~97.6%) in 17 min after elution with HEMWat 4/1/4/1 (*v*/*v*/*v*/*v*) (Figure S1).

Therefore, it can be noticed that the shortest time for the purification of rutamarin was achieved in the latter separation, whereas the first one gave a slightly higher amount of the target compound, but in a 2.4-folds longer separation time. Nevertheless, the highest purity of rutamarin was observed for the batch separation with HEMWat 3/1/3/1 (*v*/*v*/*v*/*v*). Furthermore, in order to offer a perspective about the methods’ efficiency, rutamarin was quantified by HPLC–DAD in the dichloromethane root extract of *R. graveolens*. It was found that the extract contained 5.33 ± 0.24 mg rutamarin/100 mg dwextract. Depending on the biphasic solvent system, high-purity rutamarin can be obtained from the crude extract with a yield of 40–53%.Clearly, it could be concluded that LLC can be efficiently used for the one-step purification of rutamarin from *R. graveolens*. The structure of rutamarin was elucidated by comparing the spectroscopic data (Figures S2–S6) with those reported by Zhang et al. [22].

### 2.2. In Vitro Study: hMAO-A and hMAO-B Inhibitory Activity

In the present study, the inhibition potencies of rutamarin, a dihydrofuranocoumarin, and its sourced *R. graveolens*. dichloromethane extract against *h*MAO-A and *h*MAO-B enzymes were evaluated by an in vitrofluorometric method. Table 1 presents the *h*MAO-A and *h*MAO-B inhibitory potencies of rutamarin and the crude extract. In the case of rutamarin, a weak inhibition against *h*MAO-A was observed; however, the crude extract showed a significant inhibition (88.22%). In contrast, both compound and extract indicated higher inhibition profile towards *h*MAO-B isoform. Rutamarin showed a higher selectivity for *h*MAO-B than *h*MAO-A, which was almost four times higher than the crude extract.

To date, the CNS activity of rutamarin has not been evaluated neither by in vitronorin vivoassays. A few bioactivities, like antiviral or cytotoxic have been reported [23,24]. However, one in silicostudy showed that rutamarin has a selective activity to the cannabinoid CB2 receptor (*K_i_* = 7.4 μM) [19]. The activity of furanocoumarins in the CNS is the subject of many current studies. Among linear furanocoumarins, the most investigated compounds are those substituted at C-5 or C- 8, such as imperatorin, xanthotoxin, bergapten or oxypeucedanin. Furanocoumarins have modulating properties of GABA_A_ benzodiazepine receptor activity, inducing sedative, anticonvulsant and anxiolytic effects [25]. On the other hand, several furanocoumarins showed moderate to potent inhibitory activity against mice MAOs [9]. Other naturally occurring coumarins tested against *h*MAO activity are primarily coumarins like, lacinartin, auraptene and umbelliferone. Lacinartin was isolated from the methanol extract of *Zanthoxylum schinifolium* Siebold&Zucc. stems (Rutaceae). In in vivo experiments, it showed higher affinity to *h*MAO-A compared to the *h*MAO-B isoform [11]. Further simple coumarins, such as auraptene and umbelliferone were isolated from the aerial methanol extract of *Dictamnus albus* L. (Rutaceae). Auraptene showed a slight selective inhibitory effect against *h*MAO-B (IC_50_ = 0.5 µM) compared to *h*MAO-A (IC_50_= 1.3 µM). On the other side, umbelliferone was highly selective against *h*MAO-B than *h*MAO-A, in a concentration dependent manner, with IC_50_ values of 0.6-µM and 34.6 µM, respectively [10]. Monankarins A–D are naturally occurring pyranocoumarins, which exhibited moderate activity against mice MAOs. Among them, the most potent activity was shown by monankarin C with IC_50_ = 10.7-µM and monankarin A with IC_50_=15.5-µM [12].

### 2.3. In Silico Study: Molecular Mechanisms of Rutamarin Interactions with hMAO-B

Knowing that naturally occurring coumarins are MAO-A and MAO-B inhibitors, the benzo-γ-pyrone nucleus was an object of many modifications, with the aim to find the most optimal structure for a selective inhibition. Coumarins bearing a benzyloxy substituent at C-7, such as 7-(3-chlorobenzyloxy)-4-(methylamino)methyl-coumarin,7-(3-chlorobenzyloxy)-4-carboxaldehyde–coumarin or 7-[(3,4-difluorobenzyl)oxy]-3,4-dimethylcoumarin, were shown to be effective *h*MAO inhibitors [26,27]. Further investigations of these derivatives revealed that a methylamino-methyl group at position 4 improved the solubility and pharmacokinetic properties [27]. In addition, substitution in position 3 of the coumarin nucleus modulated *h*MAO-B inhibitory activity. Substitution of aryl moiety in position 3 was based on the biologic properties of *trans*-resveratrol. The resveratrol-coumarin hybrids showed high selectivity for *h*MAO-B, with differences depending on the nature and position of the substituents at the phenyl rings. Preferred substituents at the 3-arylring were—OCH_3_, -CH_3_ and—Br, with the orientation decreasing in the order *meta* > *para* > *ortho*. In addition to MAO inhibitory activity, 3-arylderivatives are also potent acetylcholinesterase (AChE) inhibitors [8]. Another synthetic group of new coumarin–dithiocarbamatehybrids were designed and synthesized using substitution at C-7 of the coumarin nucleus. One derivative was identified as the most potent *h*MAOs inhibitor, with IC_50_values of 5.85±0.18 µM for *h*MAO-A and 0.10±0.02 µM for *h*MAO-B. Thiophen-2-yl, thiophen-3-yl or indol-3-ylcoumarin derivatives with methoxy groups at either position 6 or 7 on the(1H)-benzopyran structure resulted in inhibition of the *h*MAO-B at nanomolar concentrations [28]. The influence of different moieties substituted at the coumarin nucleus was investigated by Huang et al. [29]. Incorporating the bulky cyclohexyl at 3,4-positions led to a reduction of the efficacy against *h*MAO-B isoforms. Addition of small groups with another—CH_3_ or—Cl substituent at position 3 and cyclopentenyl at the 3,4-positions improved *h*MAO-B inhibitory activity, compared to the corresponding monosubstituted homolog. The authors also compared the non-coumarin scaffold (2-quinolinone) compounds and coumarin derivatives and suggested that the coumarin nucleus is required to obtain reasonable MAO-B inhibitory activity [29]. Additionally, coumarin 3-phenylcarboxamide scaffolds and chromone derivatives were modified by introduction of 6-methyl or 6-methoxy substituents. This change showed higher inhibition potency in 6-methylcoumarin derivatives compared to the 6-methoxy analogs. Furthermore, 6-methyl or 6-methoxy on the chromone scaffold had no noteworthy influence on MAO-B inhibitory activity [30].

Despite the wide range of data about the activity of compounds based on the primary coumarin ring described above, the information about dihydrofuranocoumarins in the context of MAOs inhibition are limited. Nevertheless, some studies showed significant neuroprotective properties of two dihydrofuranocoumarins. Marmesin and nodakenetin isolated form *Angeli*ca *gigas* Nakai root (Apiaceae) exhibited significant neuroprotective against glutamate-induced toxicity and showed 50% effective concentrations between 0.1 and 1-µM [31]. Both compounds were found to be active AChE inhibitors with almost the same IC_50_values—0.067-µM and 0.068 µM, respectively [32]. Nodakenin—the aglycone of nodakenetin—reversed scopolamine-induced cognitive impairments in the passive avoidance test and the Y-maze test (*p* < 0.05) and reduced escape-latency during training in the Morris water maze test [33]. These findings suggest that dihydrofuranocoumarins are an interesting, but not fully examined class of coumarins.

In the current study, docking simulations were performed to speculate on the possible molecular mechanism of interactions between MAO-B and rutamarin. The molecular model of *h*MAO-B co-crystalized with7-(3-chlorobenzyloxy)-4-carboxaldehyde–coumarin was used as a target structure [27]. The ligand binding site of this model (PDBid: 2V60.pdb) was occupied by the coumarin analog inhibitor, structurally similar to rutamarin molecule. Molecular models of (*R*)-rutamarin and (*S*)-rutamarin were docked into the binding pocket with MVD v.6.0. simulation package; the template docking mode used the co-crystalized inhibitor molecule as a template.

**Figure 2:**
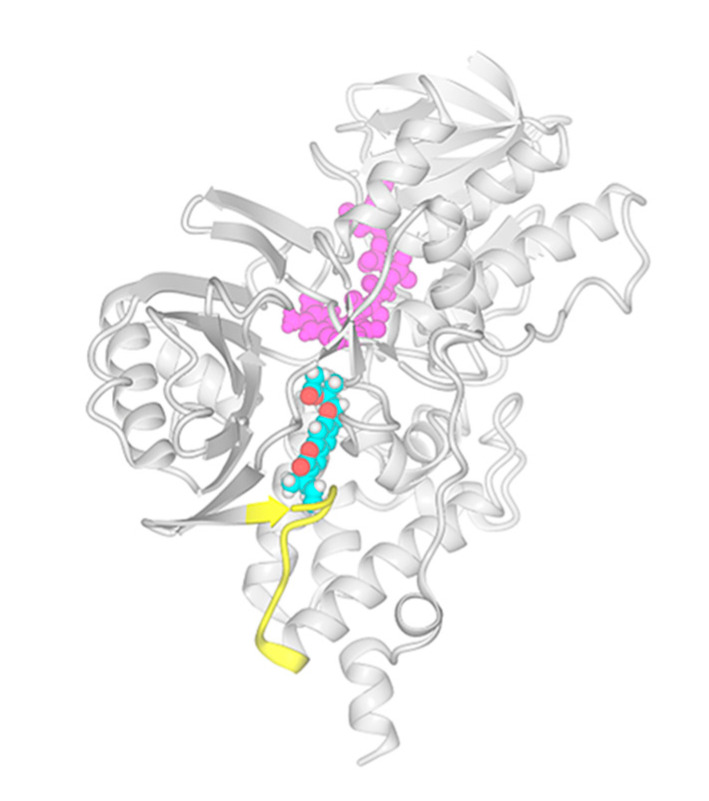
Monomer of *h*MAO-B molecule with lowest energy pose of (*S*)-rutamarin molecule docked into the binding cavity. Ribbon diagram of overall protein structure in gray, the loop formed by residues 99–109, which admits ligands into the active site is highlighted in yellow. The FAD cofactor molecule is colored in magenta and while the (*R*)-rutamarin is presented in ball mode with atom color coding mode: carbon—cyan, oxygen—red and hydrogen—white).

Docking results suggested that rutamarin could accommodate the *h*MAO-B binding pocket in a similar manner as the template coumarin analog co-crystalized in this structure (Figure 2). However, there was a significant difference in the docking poses simulated for the two enantiomers. The stereochemistry of (*R*)-rutamarin allowed the molecule to assume a similar orientation to that of the template molecule; the root mean square deviation (RMSD) measure comparing the location of common coumarin rings was calculated as 2.09Å. On the other hand, (*S*)-rutamarin encountered steric restriction within the shape of the binding site, which prevented it to assume the same orientation as the (*R*)-enantiomer. As a result, the (*S*)-rutamarin occupied the same area of the pocket, but its orientation was reversed in respect to the template molecule; the RMSD measure for coumarin rings belonging to (*S*)-rutamarin and the template was 5.77Å. The comparison of the docking score values generated in simulations for the two enantiomers suggested that (*S*)-rutamarin binds stronger to the *h*MAO-B binding cavity than (*R*)-rutamarin; MolDockscore values calculated in MVD package were −142.04 vs. −110.15, respectively and Docking score values −501.73 vs. −474,7, respectively. Detailed inspection of the two complexes indicated that the pose of (*S*)-rutamari nmay be additionally stabilized by two hydrogen bonds; one connects the—OH moiety of Y326 residue with the oxygen atom of the chromene ring, while the second interaction occurs between the—OH moiety of Y396 residue and the ester group of the ligand molecule. Simulated orientation of (*R*)-rutamarin structure within the binding site is not optimized to exercise hydrogen bond interactions with these two residues.

**Figure 3:**
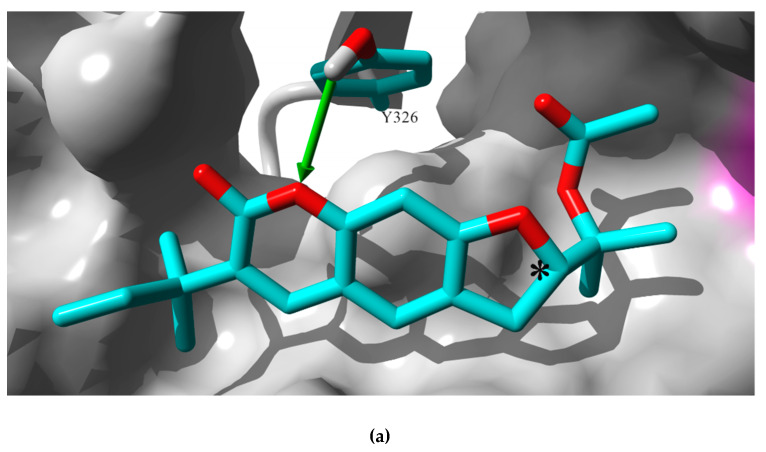

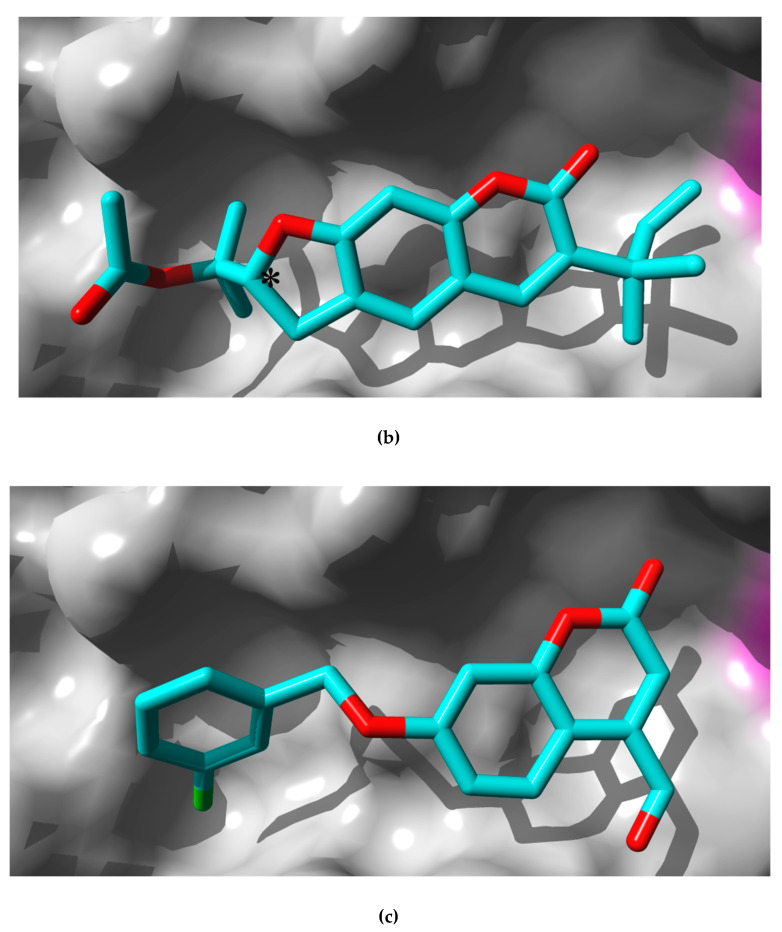
Comparison of docking orientation of (*S*)-rutamarin molecule (**a**) and (*R*)-rutamarin molecule (**b**) with 7-(3-chlorobenzyloxy)-4-carboxaldehyde–coumarin, the template co-crystalized with the binding site of the target structure (**c**). Molecules in atom color code stick mode are depicted on the surface of the binding site (gray); the FAD cofactor as a part of the catalytic center is located on the right hand side of each figure (magenta surface); asterisk denotes the carbon atom that makes the center of chirality in rutamarin structure. In (**a**), atoms of the Y326 and key hydrogen bond with (*S*)-rutamarin molecule (green arrow) are shown.

Figure 2 presents the lowest energy pose of the (*S*)-rutamarin molecule docked to the *h*MAO-B monomer structure, while Figure 3 provides the comparison of orientations within the binding pocket for (*S*)-rutamarin, (*R*)-rutamarin and the template molecule, 7-(3-chlorobenzyloxy)-4-carboxaldehyde–coumarin. Taking into consideration that (*S*)-rutamarin is the naturally occurring enantiomer, whereas (*R*)-rutamarin is the one obtained through semisynthetic reactions [22], the affinity of rutamarin isolated in the current study from *R. graveolens* for *h*MAO-B in the in vitro assay is strongly correlated with the in silico data.

## 3. Materials and Methods

### 3.1. Chemicals

Analytical grade *n*-hexane, ethyl acetate, dichloromethane and methanol were purchased from POCh (Gliwice, Poland), whereas LC grade methanol, acetonitrile and formic acid were provided by J.T. Baker (Deventer, Netherlands). Fluorometric monoamine oxidase A/B inhibitor screening kit was purchased from Biovision® (San Francisco, CA, USA). These kits include ready-to-use solutions, such as enzyme assay buffer solutions, Oxired Probe solutions in DMSO, MAO A/B enzymes (lyophilized), MAO A/B substrates (lyophilized), developers (lyophilized), inhibitor control (selegiline and clorgiline) (lyophilized) solutions.

### 3.2. Plant Material and Extraction

The aerial parts of *Ruta graveolens* L. (Rutaceae) were collected in summer 2016 in the Medicinal Plant Garden, Medical University of Lublin (Poland) and identified by Mrs. Krystyna Dąbrowska from the Botanical Garden of UMCS Lublin, Poland. A voucher specimen (200/16) is deposited in the Department of Pharmacognosy, Medical University of Lublin (Poland). The air-dried and grounded plant material (40 g) was subjected to ultrasound-assisted extraction for 30 min at room temperature with dichloromethane (3 × 160 mL). After combining all extracts and solvent removal, 1.15 g of crude extract (yield: 2.9%) resulted.

### 3.3. Liquid–Liquid Chromatographic Separation

The LLC separation of rutamarin was performed on a spectrum high-performance countercurrent chromatograph (Dynamic Extractions, Slough, UK) equipped with two bobbins that fitted both the analytical (22 mL, 0.8 mm i.d., 1-mL loop) and semipreparative (136 mL, 1.6 mm i.d.,6-mL loop) PTFE coils. The apparatus was connected to an Alpha 10 pump and a Sapphire UV detector (ECOM, Prague, Czech Republic). Several *n*-hexane-ethyl acetate–methanol–water (HEMWat) mixtures (5/2/5/2, 3/1/3/1 and 4/1/4/1 *v*/*v*/*v*/*v*) were screened in a series of shake-flask experiments, following the procedures described in [34]. The suitability of each biphasic solvent system was evaluated on the basis of the partition coefficient of rutamarin (*P_rut_*) values. Next, the selected solvent system was prepared in a separation funnel and thoroughly equilibrated after shaking at room temperature. The upper and lower phases were labeled in two different recipients and both degassed for 10 min prior to analyses. LLC experiments were performed as follows: the semipreparative coil was initially filled with the upper organic stationary phase. Then, the rotation of the apparatus was set to 1600 rpm (to achieve the maximum centrifugal force of 240 × g) and the lower aqueous mobile phase was pumped at a flow-rate of 6 mL/min (reversed-phase mode, head-to-tail mode). After the hydrodynamic equilibrium was attained (as indicated by the emergence of the mobile phase front), pulse injections were performed, each time with 100 mg of crude dichloromethane extract of *R. graveolens* L. dissolved in 6 mL of the corresponding solvent system. The UV spectrum was recorded at 335 nm and 1 min fractions were collected. The purity of each fraction was next assessed by HPLC–DAD analyses.

### 3.4. HPLC–DAD Analysis

The HPLC–DAD analysis of the crude extract and all collected fractions was performed on a Shimadzu HPLC (Tokyo, Japan) equipped with a degasser (DGU-20 A 3R), binary pump (LC-20AD), autosampler (SIL-20 AHT) and DAD detector (SPD-M20 A). The separations were carried out on a Zorbax Eclipse XDB C18 stainless-steel (250 × 4.6 mm, 5 μm) column, following the conditions described by Luca et al. [34]. Rutamarin content in the crude extract was determined by using the same method with the help of a calibration curve plotted with the corresponding standard (linearity range: 25–250 μg/mL, regression equation: y = 19373x − 31740,regression coefficient *R^2^*= 0.9998).

### 3.5. Structure Elucidation

Rutamarin was identified by HRESIMS and NMR analyses. The HPLC–DAD-ESI-Q-TOF-MS/MS was performed on an Agilent 1200 HPLC (Agilent Technologies, Santa Clara, CA, USA) equipped with an auto-sampler (G1329B), binary pump (G1312C), thermostat (G1316A), DAD (G1315D) and ESI-Q-TOF-MS (G6530B). The separation was carried out on a Phenomenex Gemini C18 (100×2mm, 3μm) column, according to a method previously described by Luca et al. [13]. ^1^H-NMR, ^13^C-NMR, COSY, HSQC, HMBC and ROESY analyses were performed on a Bruker Avance Neo 600MHz NMR spectrometer (BrukerBioSpin, Rheinstetten, Germany) equipped with a QCI 5 mm Cryoprobe and a SampleJet automated sample changer. CD_3_OD (δ_H_3.31;δ_C_49.0) was used as internal standard for ^1^H- and ^13^C-NMR.

**Rutamarin** UV (MeOH): 335 nm; HRESIMS: *m*/*z*357.1718 [M+H]^+^ (calcd. for C_21_H_25_O_5_^+^, *m*/*z* 357.1696, ∆ = 6.09 ppm); MS/MS (20 eV): 315.1611, 301.1082, 297.1457, 273.1107, 259.0951, 255.1015, 241.0842, 223.0779; ^1^H-NMR (CD_3_OD, 600 MHz): δ 1.46 (6H, s, H-2’’, H-3’’), 1.54 (6H, s, H-2’, H-3’), 1.92 (3H, s, H-5’), 3.25 (1H, ddd, *J*=15.8, 7.5, 1.1 Hz, H-3b), 3.31 (1H, ddd, *J*=15.8, 9.2, 1.1 Hz, H-3a), 5.06 (3H, m, H-2, H-5’cis, H-5’trans), 6.17 (1H, dd, *J*=17.5, 10.7 Hz, H-4’’), 6.69 (1H, s, H-9), 7.41 (1H, d, *J*=1.4 Hz, H-4), 7.73 (1H, s, H-5); ^13^C-NMR (CD_3_OD, 151 MHz) δ 21.5 (C-3’), 22.0 (C-2’), 22.1 (C-5’), 26.6 (C-2’’, C-3’’), 30.4 (C-3), 41.2 (C-1’’), 83.7 (C-1’), 90.1 (C-2), 97.4 (C-9), 112.4 (C-5’’), 114.5 (C-4a), 124.9 (C-4), 126.3 (C-3a), 131.4 (C-6), 140.3 (C-5), 146.9 (C-4’’), 155.9 (C-8a), 162.2 (C-7), 164.2 (C-9a), 172.1 (C-4’). MS/MS fragmentation patterns of rutamarin are proposed in Figure S2.

### 3.6. In Vitro Enzymatic Activity Assay

The *h*MAO-A/B enzymes inhibitors screening methods were evaluated according to Biovision^®^’s fluorometric assays guidelines with small modifications. These procedures are based on the fluorometric detection of H_2_O_2_ which is one of the byproducts generated during the oxidative deamination of tyramine, the *h*MAO substrate. The OxiRed™, a very sensitive and stable probe was used for H_2_O_2_. *h*MAO-A/B inhibitor screening protocol: The experiments were performed in black flat-bottom 96-well. In each well, 50 μL of *h*MAO-A/B enzyme solution mixed with 10 μL of the crude extract/rutamarin/positive control was incubated for 10 min at 25 °C (for MAO-A) or at 37 °C (for MAO B). Then, to start the enzymatic reaction, 40 μL of *h*MAO-A/B substrate, tyramine solution was added into the each well. The mixtures were incubated for 30 min at the same temperatures mentioned above. The fluorescence was kinetically measured using 535/587 nm (excitation/emission) at 5-min intervals. DMSO was used as solvent control. Clorgiline and selegiline were used as positive controls (selective inhibitors of *h*MAO-A and *h*MAO-B, respectively).

### 3.7. Docking of Rutamarin into hMAO-B Binding Site

Docking simulations were performed to the target molecular model representing human MAO- B protein co-crystalized with 7-(3-chlorobenzyloxy)-4-carboxaldehyde–coumarin [24]. Molecular models of (*R*)-rutamarin and (*S*)-rutamarin were constructed in HyperChem 6.05 software while MVD v. 6.0. simulation package was used for docking simulations. In each simulation, 10 docking runs were performed where co-crystalized present in the binding pocket served as a template. The lowest energy pose was selected using the MolDockscore function ranking, and the results presented in Section 2.3. Figure 2 and Figure 3 were prepared using the YASARA 19.5.23. package.

## 4. Conclusions

This is the first time that a rapid and effective isolation method for rutamarin is presented. Liquid–liquid chromatography was successfully used for a one-step purification of rutamarin, yielding the target component with purity higher than 97% (HPLC–DAD)—and in quantities that allowed further biologic investigations. In vitro enzymatic studies showed that rutamarin is a promising and selective *h*MAO-B inhibitor, which was confirmed by in silico studies. However, further studies on specific enantiomers of rutamarin are needed. These results also shed new light on dihydrofuranocoumarin as a potential interesting natural product scaffolds to be considered for the development of drug leads in the treatment of CNS disorders.

## Figures and Tables

**Table 1:** Human monoamine oxidase (*h*MAO)-inhibitory activities of rutamarin and *R. graveolens* L. dichloromethane extract.

Sample	Concentration	%INH(*h*MAO-A)	%INH(*h*MAO-B)	SI ^1^
*Rutamarin*	6.17 μM	25.15	95.26	3.788
*R. graveolens* extract	9.78 mg/mL	88.22	89.98	1.020
Reference selective MAO-A or MAO-B inhibitors				
Selegiline	5.34 μM	–	99.07	–
Clorgiline	3.67 μM	99.29	–	–

^1^SI (*h*MAO-B selectivity index) =%INH(*h*MAO-B)/%INH(*h*MAO-A).

## Supplementary Materials

Supplementary materials can be found at https://www.mdpi.com/1420-3049/25/11/2678/s1. Figure S1: Chromatograms of CCC separations rutamarin from a crude dichloromethane extract of *Ruta graveolens* L. Figure S2: MS/MS fragmentation patterns proposed for rutamarin. Figure S3: ^1^H NMR spectrum of rutamarin in CD_3_OD. Figure S4: COSY NMR spectrum of rutamarin in CD_3_OD. Figure S5: ^13^C-DEPTQ NMR spectrum of rutamarin in CD_3_OD at 151 MHz. Figure S6: Edited-HSQC NMR spectrum of rutamarin in CD_3_OD. Figure S7: HMBC NMR spectrum of rutamarin in CD_3_OD. Figure S8: ROESY NMR spectrum of rutamarin in CD_3_OD.

## Abbreviations

AD	Alzheimer’s disease
CNS	Central nervous system
H_2_O_2_	Hydrogen peroxide
HEMWat	*n*-hexane/ethyl acetate/methanol/water
LLC	liquid–liquid chromatography
MAOs	Monoamine oxidases
PD	Parkinson’s disease
WHO	World Health Organization
