# Supplementary material for: Rutamarin: Efficient Liquid–Liquid Chromatographic Isolation from Ruta graveolens L. and Evaluation of Its In Vitro and In Silico MAO-B Inhibitory Activity
Source: Molecules. 2020 Jun 9;25(11):2678. doi: 10.3390/molecules25112678 (PMC7321355; doi:10.3390/molecules25112678)
Supplement: Supplementary file 1 [file molecules-25-02678-s001.pdf]

molecules

MDPI

Suplementary material

Rutamarin: Efficient liquid-liquid chromatographic isolation from Ruta graveolens L. and evaluation of its in vitro and in silico MAO-B inhibitory activity

Ewelina Kozioł [1], Simon Vlad Luca [2,3], Hale Gamze Ağalar [4], Begüm Nurpelin Sağlık [4], Fatih Demirci [4,5], Laurence Marcourt [6], Jean-Luc Wolfender [6], Krzysztof Jóźwiak [7], Krystyna Skalicka-Woźniak [1,*]

[1] Independent Laboratory of Natural Products Chemistry, Department of Pharmacognosy, Medical University of Lublin, 20-093 Lublin, Poland; ewelinakoziol@umlub.pl
[2] Department of Pharmacognosy, Grigore T. Popa University of Medicine and Pharmacy Iasi, 700115 Iasi, Romania; simon-vlad.v.luca@d.umfiasi.ro
[3] Biothermodynamics, TUM School of Life and Food Sciences Weihenstephan, Technical University of Munich, 85354 Freising, Germany
[4] Department of Pharmacognosy, Faculty of Pharmacy, Anadolu University, 26470 Eskisehir-Turkey; ecz.halegamze@gmail.com (H.G.A.); bnsaglik@anadolu.edu.tr (B.N.S.); demircif@gmail.com (F.D.)
[5] Faculty of Pharmacy, Eastern Mediterranean University, 99628 Famagusta, N. Cyprus
[6] Institute of Pharmaceutical Sciences of Western Switzerland, IPSWS, University of Geneva, CMU, 1211 Geneva 4, Switzerland; laurence.marcourt@unige.ch (L.M.); Jean-Luc.Wolfender@unige.ch (J.-L.W.)
[7] Department of Biopharmacy, Medical University of Lublin, 20-093 Lublin, Poland; krzysztof.jozwiak@umlub.pl
* Correspondence: kskalicka@pharmacognosy.org

Supplementary File Content

Figure S1. Chromatograms of CCC separations rutamarin from a crude dichloromethane extract of *Ruta graveolens* L.
Figure S2 MS/MS fragmentation patterns proposed for rutamarin
Figure S3. ^{1}H-NMR spectrum of **rutamarin** in CD_3OD
Figure S4. COSY NMR spectrum of **rutamarin** in CD_3OD
Figure S5. ^{13}C-DEPTQ NMR spectrum of **rutamarin** in CD_3OD at 151 MHz
Figure S6. Edited-HSQC NMR spectrum of **rutamarin** in CD_3OD
Figure S7. HMBC NMR spectrum of **rutamarin** in CD_3OD
Figure S8. ROESY NMR spectrum of **rutamarin** in CD_3OD

a

b

c

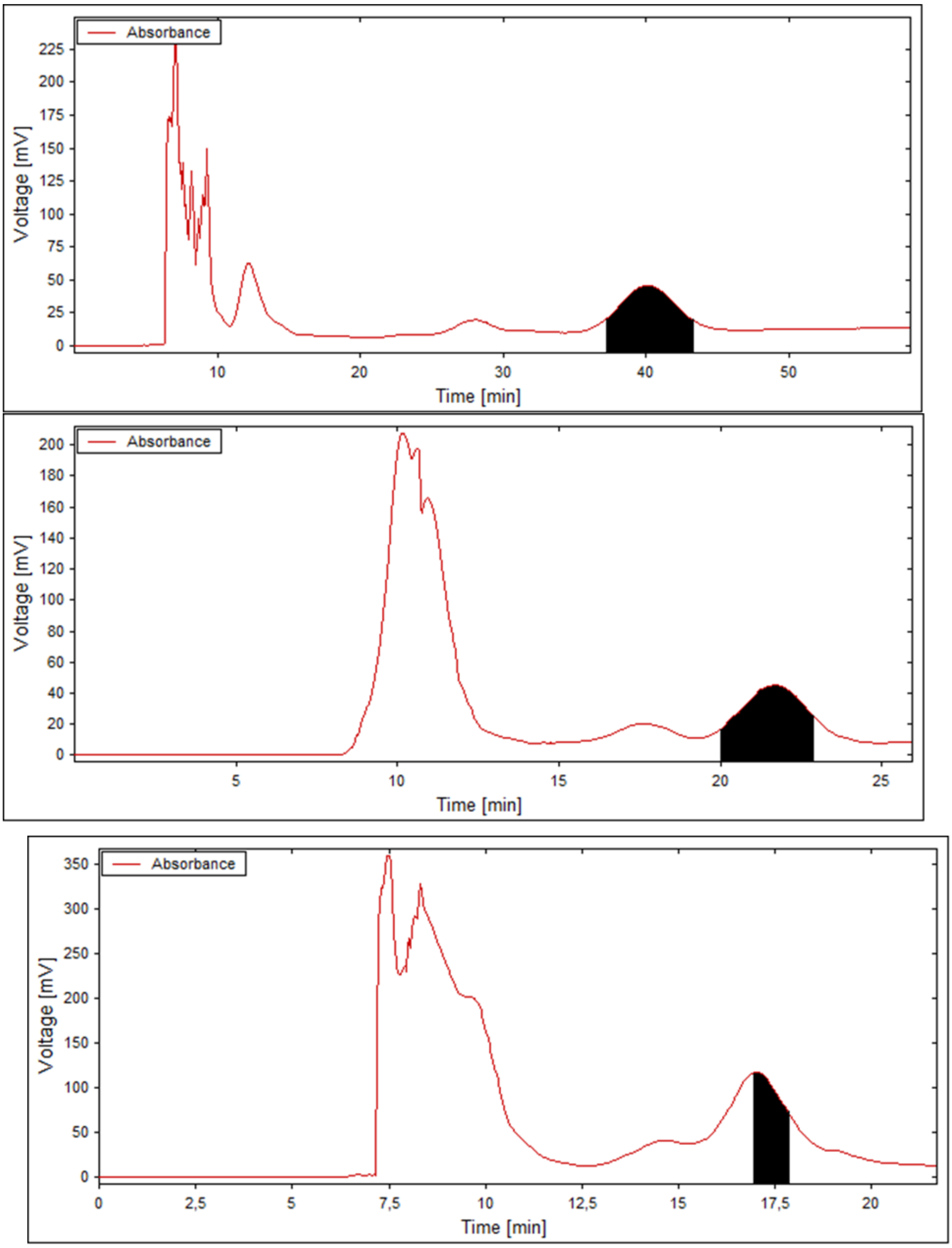

Figure S1. Chromatograms of CCC separations rutamarin from a crude dichloromethane extract of *Ruta graveolens* L. with (**a**) *n*-hexane/ethyl acetate/methanol/water (5/2/5/2, *v*/*v*/*v*/*v*) (**b**) *n*-hexane/ethyl acetate/methanol/water (3/1/3/1, *v*/*v*/*v*/*v*) and (**c**) *n*-hexane/ethyl acetate/methanol/water (4/1/4/1, *v*/*v*/*v*/*v*); upper phase as stationary phases (reversed-phase, head-to-tail mode, descending mode); flow-rate: 6 mL/min; V_{inj} = 6 mL; C_{inj} = 16.67 mg/mL; ω = 1900 rpm, S_F = 80%; UV 335 nm.

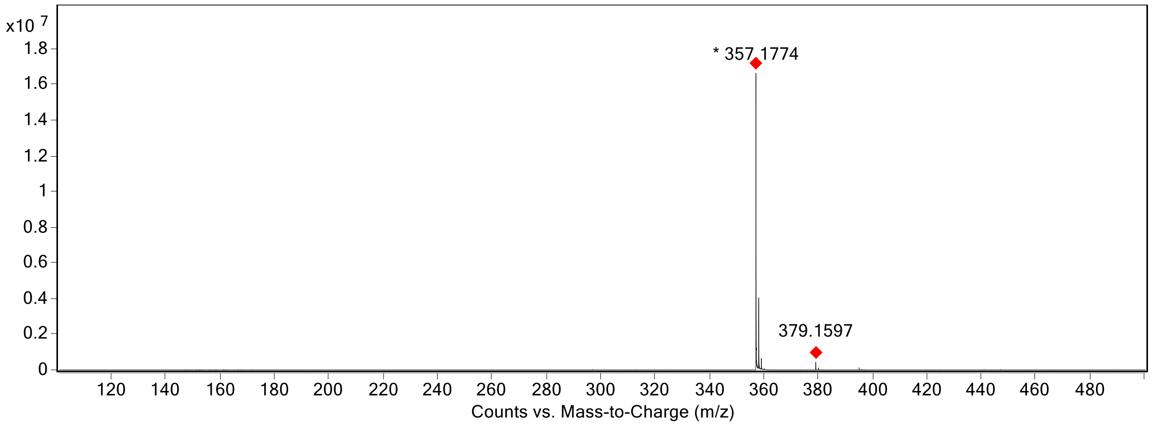

Figure S2a. HRESIMS spectrum of rutamarin.

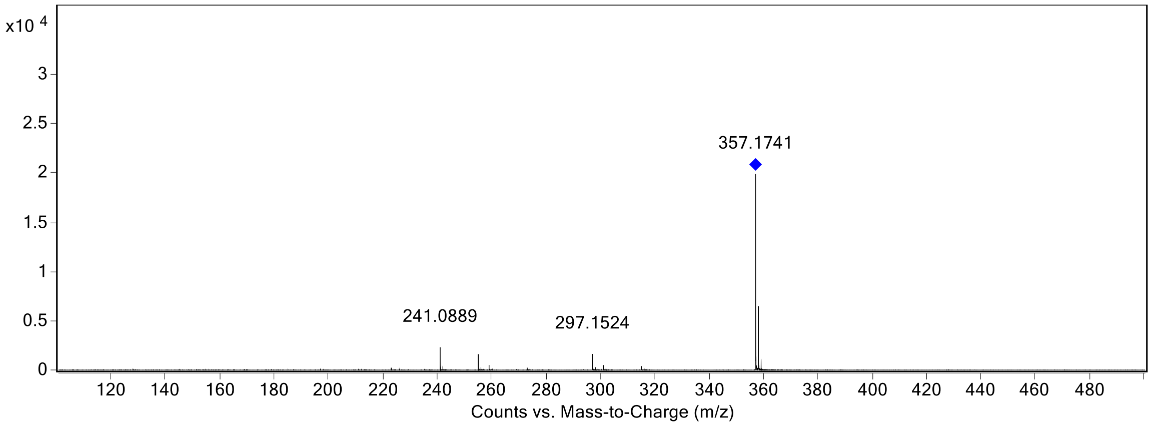

Figure S2b. MS/MS spectrum of rutamarin.

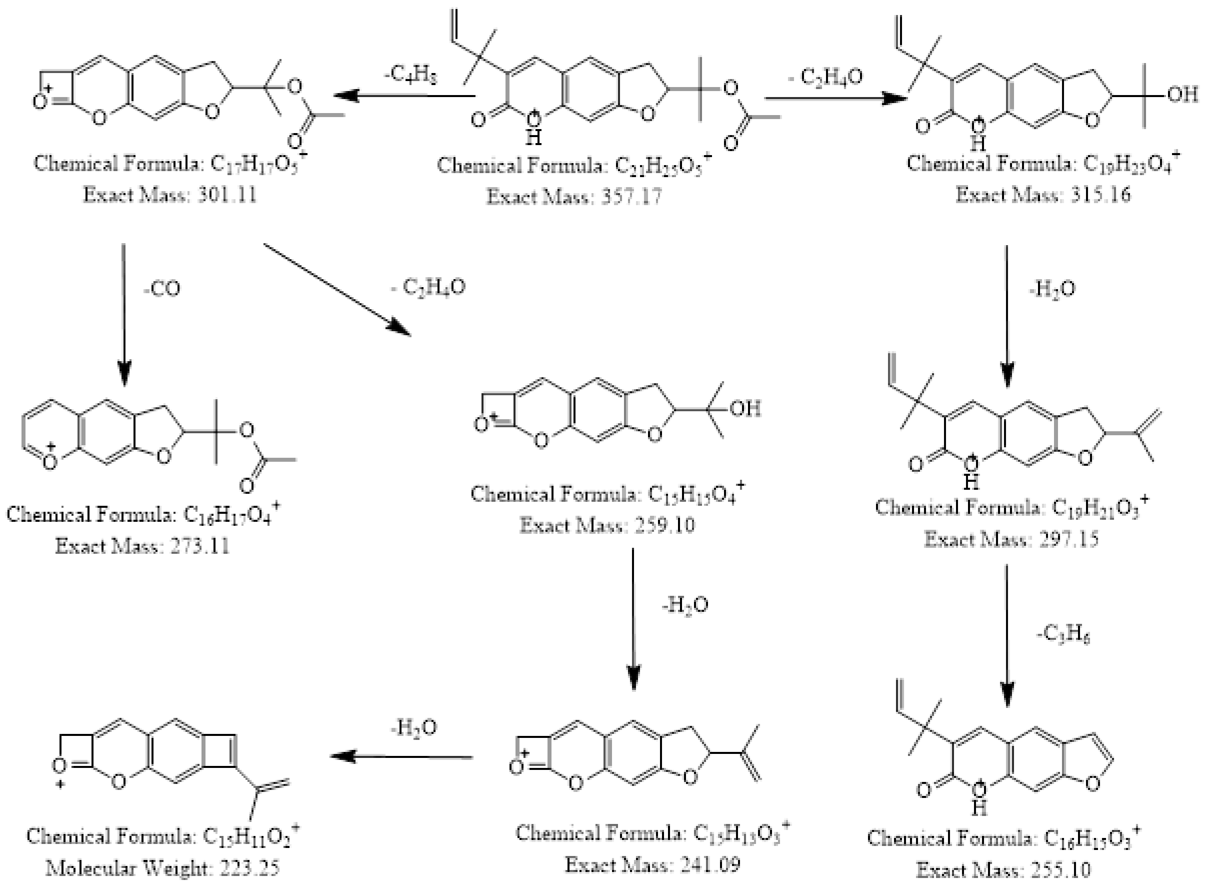

Figure S2c. MS/MS fragmentation patterns proposed for **rutamarin.**

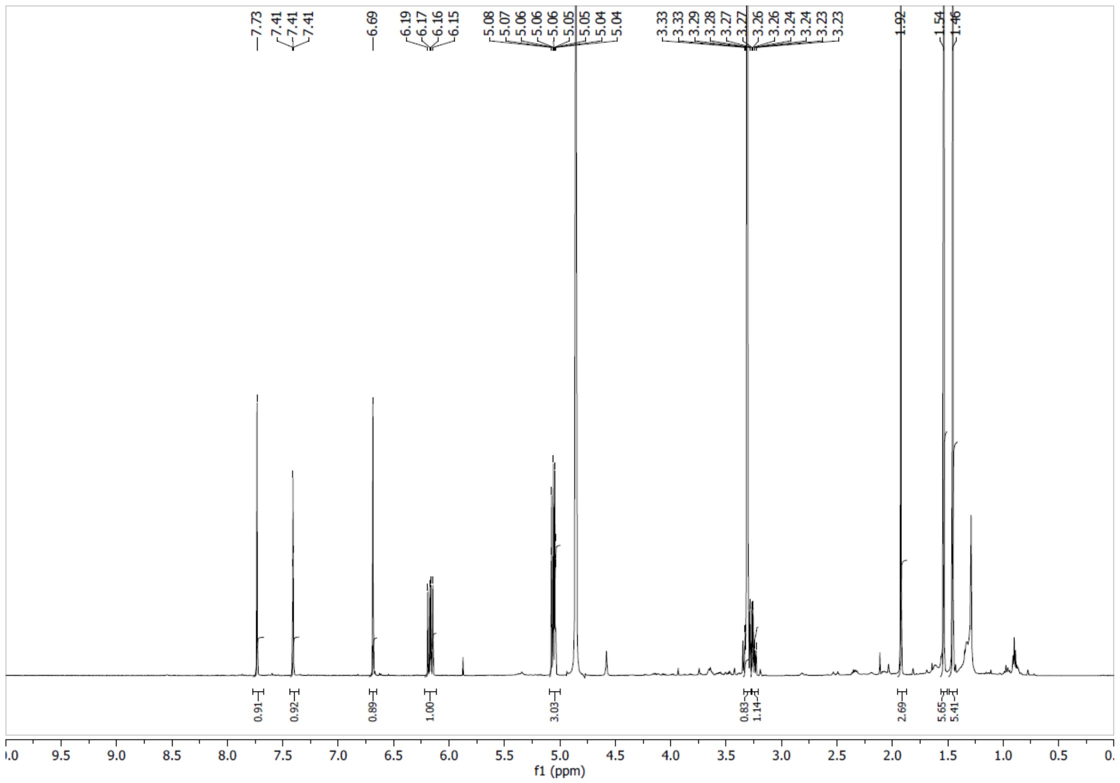

Figure S3. ^{1}H-NMR spectrum of **rutamarin** in CD_3OD.

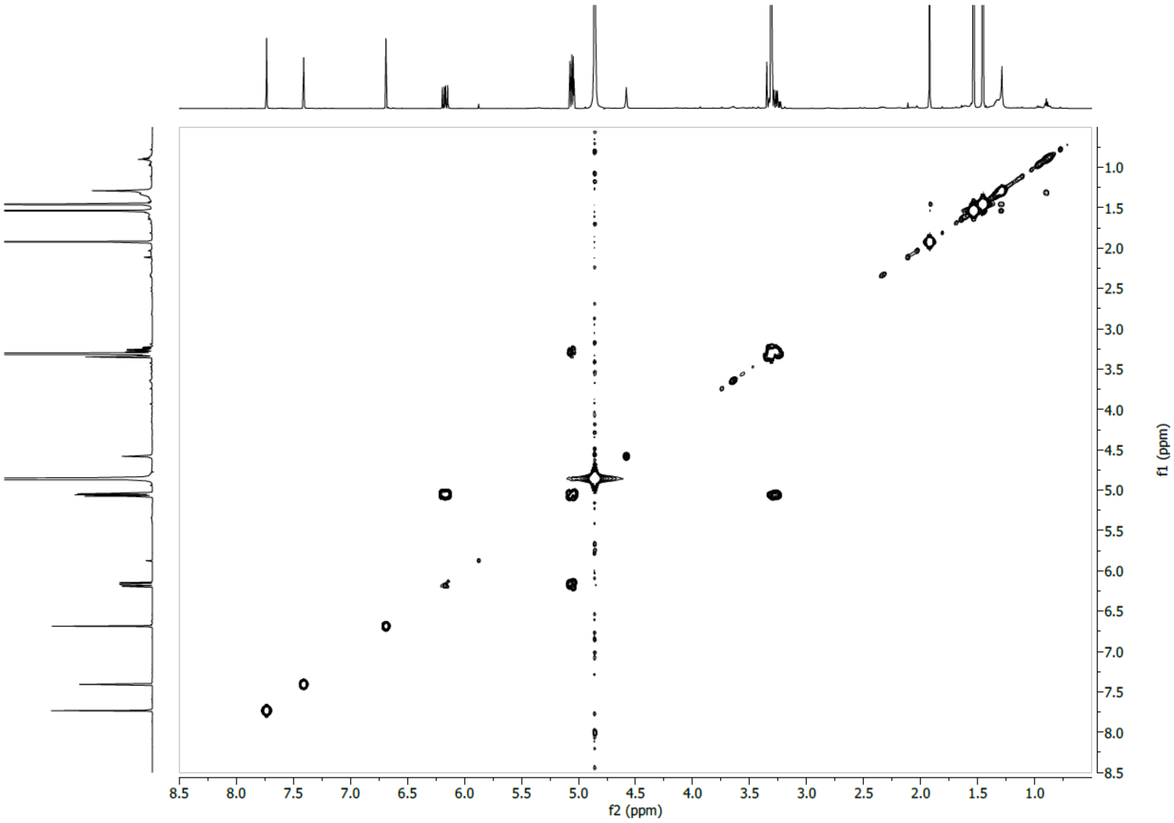

Figure S4. COSY NMR spectrum of **rutamarin** in CD_3OD.

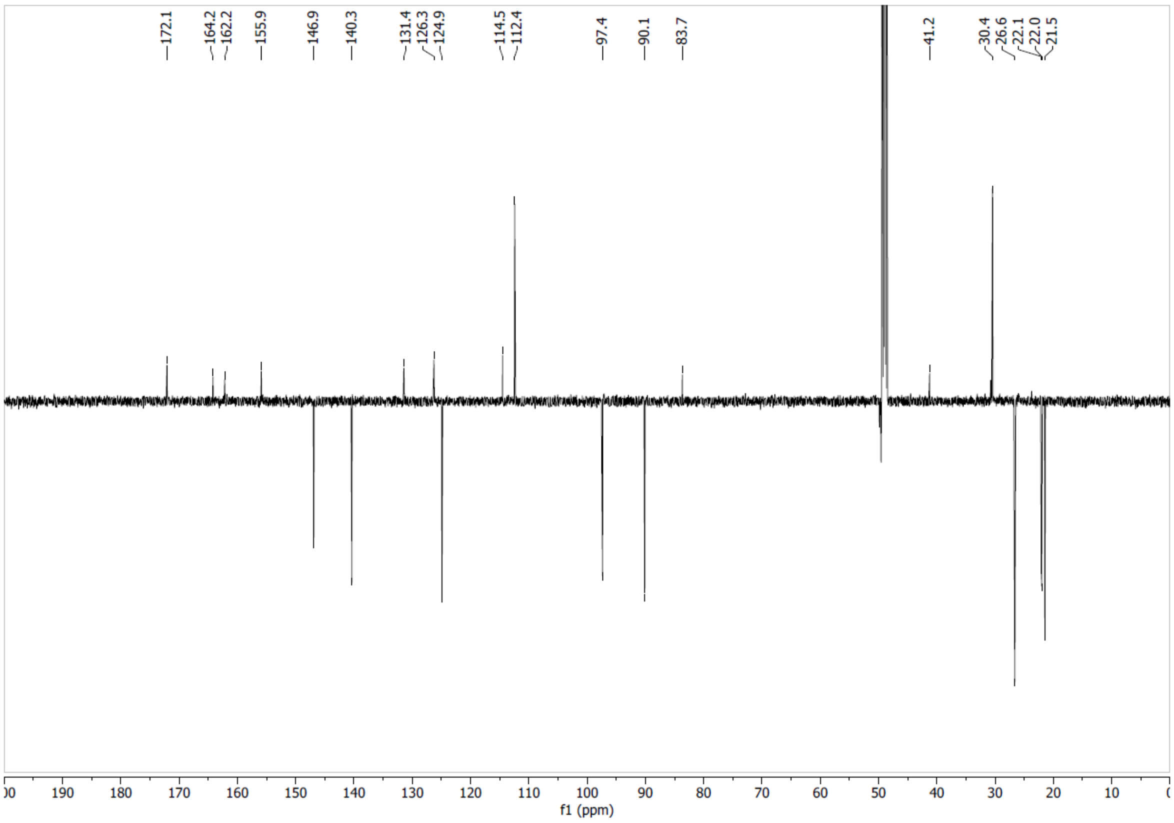

Figure S5. ^{13}C-DEPTQ NMR spectrum of **rutamarin** in CD_3OD at 151 MHz.

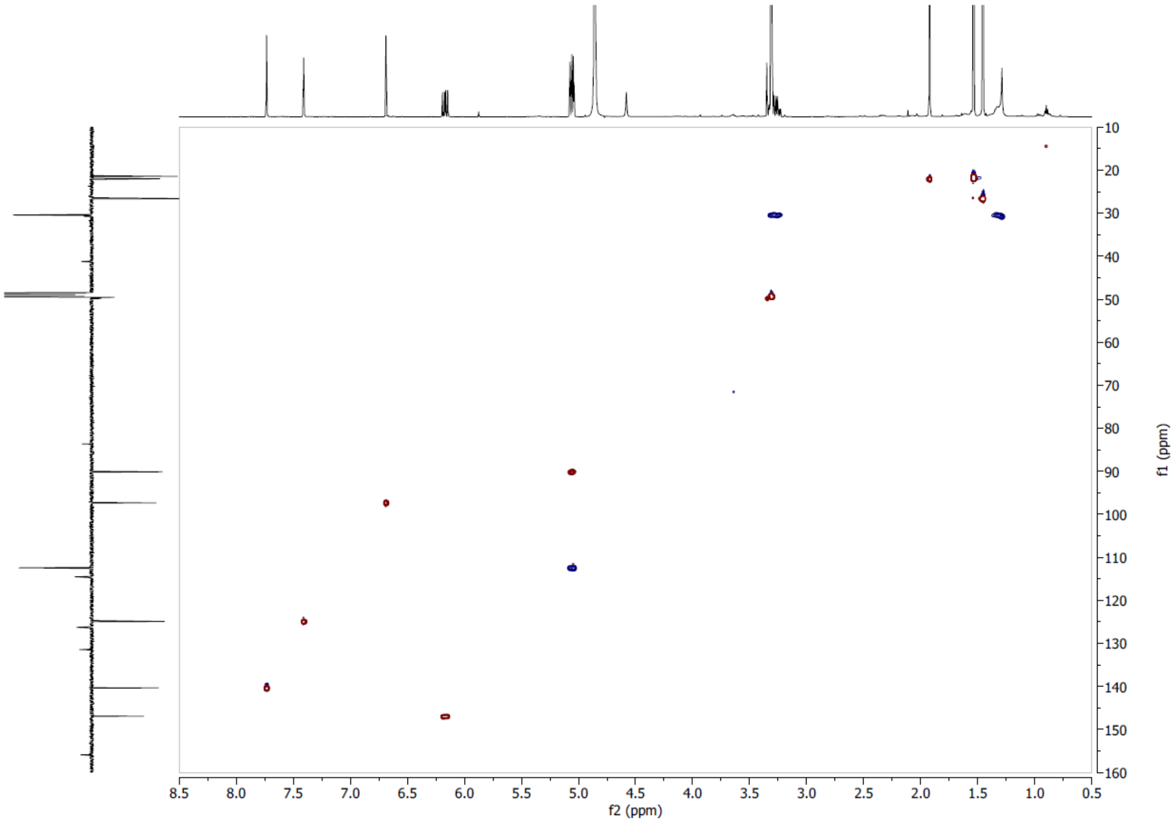

Figure S6. Edited-HSQC NMR spectrum of **rutamarin** in CD_3OD.

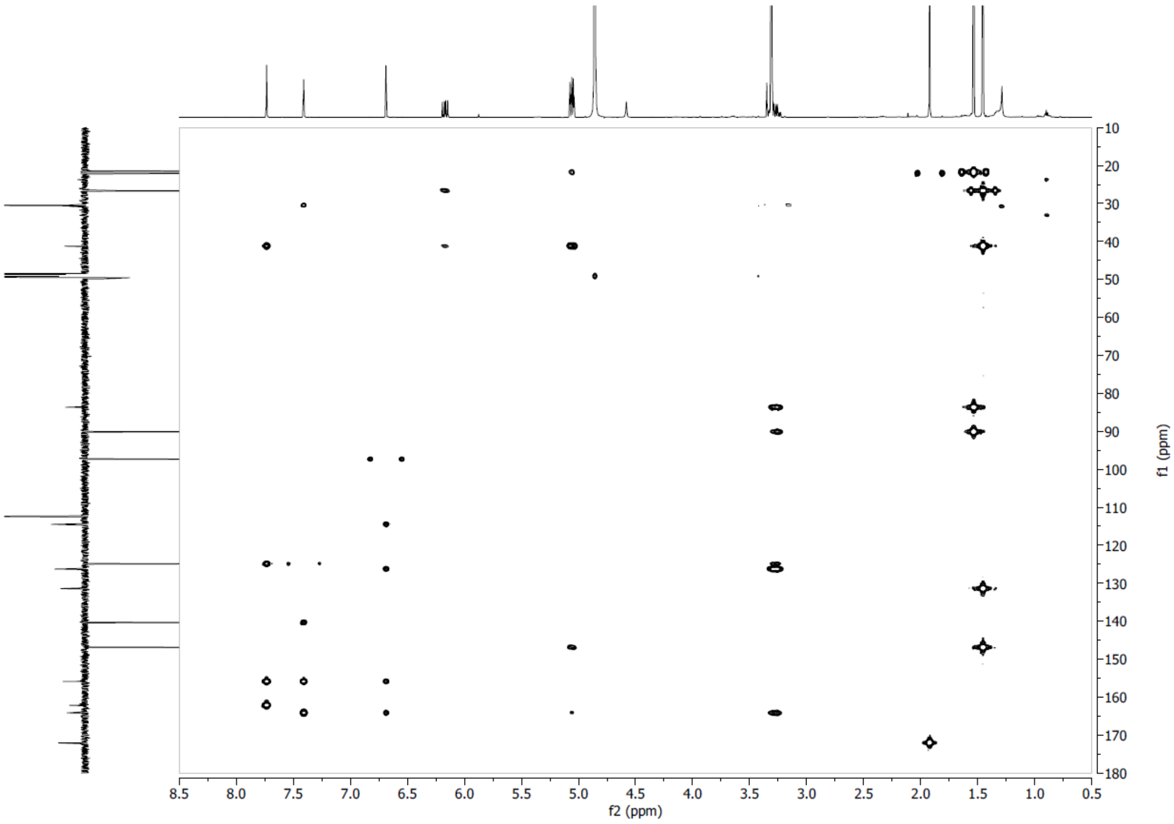

Figure S7. HMBC NMR spectrum of **rutamarin** in CD_3OD.

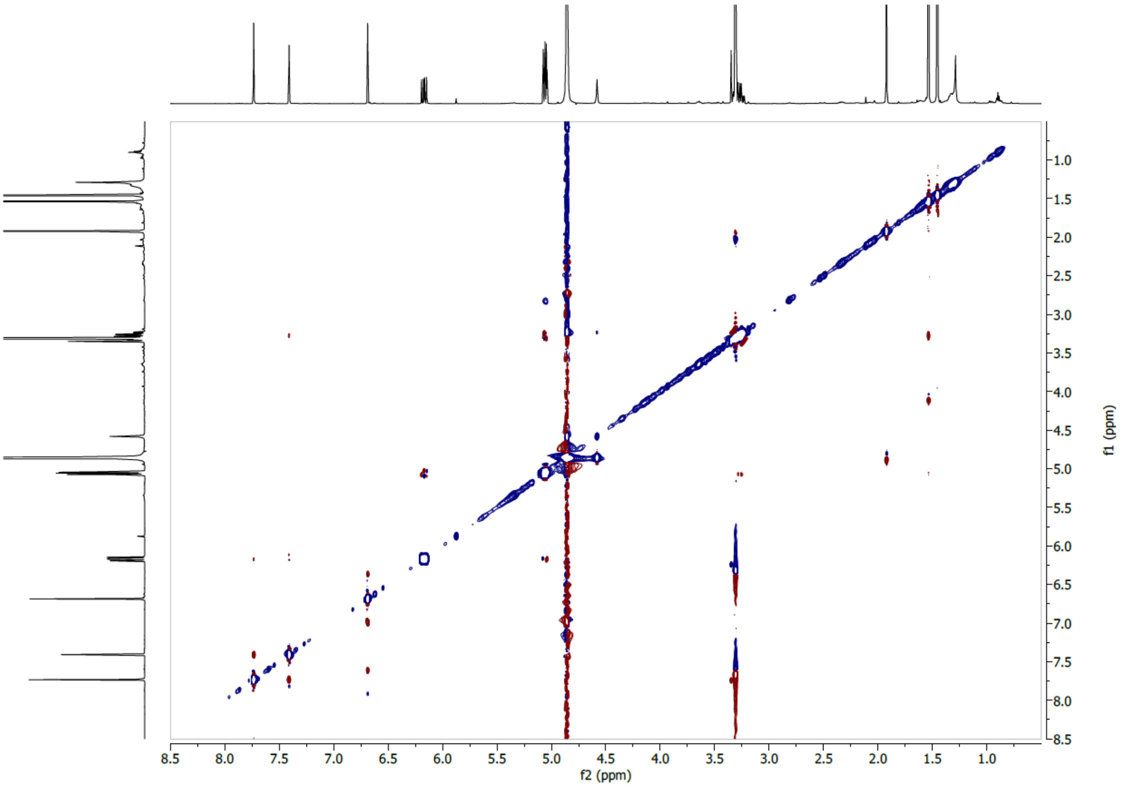

Figure S8. ROESY NMR spectrum of **rutamarin** in CD_3OD.

© 2020 by the authors. Submitted for possible open access publication under the terms and conditions of the Creative Commons Attribution (CC BY) license (http://creativecommons.org/licenses/by/4.0/).
